# Evaluation of the Macular, Peripapillary Nerve Fiber Layer and Choroid Thickness Changes in Behçet's Disease with Spectral-Domain OCT

**DOI:** 10.1155/2014/865394

**Published:** 2014-04-24

**Authors:** Mustafa Ataş, İsa Yuvacı, Süleyman Demircan, Emel Güler, Orhan Altunel, Emine Pangal, Altan Göktaş, Serap Sütbeyaz, Gökmen Zararsız

**Affiliations:** ^1^Department of Ophthalmology, Kayseri Research and Teaching Hospital, Kocasinan, Kayseri, Turkey; ^2^Department of Physical Medicine and Rehabilitation, Kayseri Research and Teaching Hospital, Turkey; ^3^Department of Biostatistics, Medical School, University of Erciyes, Turkey

## Abstract

*Purpose*. To assess the macular, choroid, and peripapillary nerve fiber layer thickness (RNFL) in Behçet's disease (BD) patients with and without ocular involvement by means of optical coherence tomography (OCT) and compare these findings with healthy controls. *Design*. Eighty patients with BD and 40 healthy controls who were followed up at the Uveitis and Retina Clinic of the Kayseri Research and Education Hospital in Turkey were enrolled in this prospective study. *Subjects and Controls*. The patients with BD were divided into two groups according to the presence of ocular involvement. Group 1 consisted of 40 eyes of 40 patients with ocular involvement and group 2 consisted of 40 eyes of 40 patients without ocular involvement. *Methods*. All of the patients and controls underwent macular, choroid, and peripapillary nerve fiber layer thickness analysis with Spectralis domain OCT (Spectralis OCT Heidelberg Engineering, Dossenheim, Germany). *Main Outcome Measures*. The differences in macular, choroid, and peripapillary nerve fiber layer thicknesses between groups were analyzed statistically. *Results*. Macular thickness was thinner in patients with BD than in the control group; this result was statistically significant (*P* = 0.05). There was no statistically significant difference in thickness between RNFL analysis of the patients with BD and control subjects. However, the BD patients with ocular involvement had statistically significant thinning in RNFL compared with BD patients without ocular involvement. Although the choroid was thicker in patients with BD than in the control group, it did not reach a statistically significant level (*P* = 0.382). *Conclusions*. BD with ocular involvement may be associated with decreased macular and RNFL thickness measured with spectral-domain OCT.

## 1. Introduction

Behçet's disease (BD) is a multisystemic chronic vasculitis characterized by recurrent uveitis, arthritis, oral and genital aphthous ulcerations, and skin lesions [1]. BD may influence almost all tissues, organs, and systems in the body, including ocular, cardiovascular, gastrointestinal, renal, pulmonary, urologic, and central nervous systems and the joints [2].

Ocular involvement occurs in approximately 70% of patients with BD and posterior segment involvement is reported in 50–93% of those patients with ocular involvement [3, 4]. The most common form of involvement is nongranulomatous anterior uveitis. Other ocular findings in BD include iridocyclitis, scleritis, episcleritis, keratitis, vitritis, vitreous hemorrhage, optic neuritis, retinal neovascularization, and chorioretinal scars. Posterior uveitis and retinal vasculitis are the main reasons for vision loss in patients with BD [3].

Posterior uveitis may affect all structures in the posterior pole of the eye, including the choroid and the retina. However, one of most common cases of posterior uveitis observed in patients with BD is necrotising vasculitis of the retina and choroid that results in vascular occlusion with subsequent ischemia and atrophy, followed by retinal and disk neovascularizations [5, 6].

Although these structural changes observed in the retina and choroid can be observed by indocyanine green angiography (ICG) and fluorescein angiography (FA), optical coherence tomography (OCT), a noninvasive, noncontact, transpupillary imaging modality that can show structural changes even in micrometer level, provides detailed information about these changes in BD with posterior segment involvement. In addition, OCT allows for reproducible measurements of choroidal and retinal thickness. To our knowledge, there are few studies in the literature that examine the thickness of the choroid in patients with BD [7–9].

There are some controversies about structures of the eye primarily affected in BD.

The aim of this study is to assess the macular, choroid, and peripapillary nerve fiber layer thickness in BD patients with and without ocular involvement by means of OCT and to compare these findings with healthy controls, so that investigating the macular, choroid, and peripapillary nerve fiber layer thickness (RNFL) would present beneficial results for a better understanding of the pathophysiology of BD.

## 2. Methods

Eighty patients with BD being followed up at the Uveitis and Retina Clinics of the Kayseri Research and Education Hospital and 40 healthy volunteers (control group) were enrolled in this study. This prospective study was performed between January 2013 and April 2013 at Kayseri Education and Research Hospital of Medicine, a tertiary referral center in Turkey. The study was approved by the institutional ethics committee (number 2013/61) and all participants signed an informed consent form to participate in the study. The study adhered to the Declaration of Helsinki.

All patients in the study fulfilled the international criteria for BD [10]. Ocular surgery, ocular trauma, glaucoma, cystoid macular edema, macular degeneration, optic atrophy, intraocular pressure (IOP) > 21 mmHg, cataracts, high spherical >±3 diopter or cylindrical >±2 diopter refractive errors, and uveitis at the time of OCT measurement were grounds for excluding patients from the study. In addition, patients who could not be examined with OCT were excluded from the study.

The patients with BD were divided into two groups according to the presence of ocular involvement. Group 1 consisted of 40 eyes of 40 patients with ocular involvement and group 2 consisted of 40 eyes of 40 patients without ocular involvement. In group 1, posterior segment involvement was present in 20 eyes of 20 patients and the remaining eyes had anterior segment involvement. There was no panuveitis in any eyes. Patients who had only a history of iridocyclitis were classified as anterior segment involvement; those who had a history of retinitis, retinal vasculitis, and/or papillitis without significant iridocyclitis were classified as posterior segment involvement. Patients who had both anterior and posterior segment involvement were classified as panuveitis. All patients enrolled in the study were in a remission/inactive phase. We defined patients who had no signs of active inflammation, such as hypopyon, macular edema, retinitis, vasculitis, papillitis, and vitritis, for a month as being in the remission/inactive phase. The control group that applied to our clinic for refractive errors consisted of 40 eyes of 40 volunteers who did not have any systemic or ocular disorders. Healthy controls were selected in accordance with the exclusion criteria: the best corrected visual acuity worse than 20/30 and spherical equivalent refractive errors of more than ±3 diopter. For a better statistical evaluation, as well as to prevent bias, only the right eyes of the BD patients and healthy control subjects were involved in the study.

We followed the review with a detailed physical examination and routine laboratory tests, including erythrocyte sedimentation rate and C-reactive proteins.

Demographic features and clinical information involving age, sex, and duration of BD were recorded. All of the individuals underwent a detailed ophthalmologic examination, including slit lamp biomicroscopic examination, measurement of best-corrected Snellen visual acuity, IOP measurements, dilated fundus examination, and fluorescein angiography (CS-60DSI, Canon, Tokyo, Japan). To assess the severity of retinal vascular leakage, the leakage was classified ranging from 0 to 3 according to a grading system used previously by other studies [11].

Grade 0 means no vascular leakage or staining, grade 1 is mild, grade 2 is moderate, and grade 3 means severe (even greater leakage with blurring of the large vessel margins).

Following the detailed ophthalmologic examination, the Spectralis domain (SD) OCT device (Spectralis OCT Heidelberg Engineering, Dossenheim, Germany) was used for the assessment. The SD-OCT assessments involved in the study were performed by the same experienced technician. The procedure was achieved without pupillary dilatation and under the same intensity of dim room lighting. The Spectralis OCT device (software version 5.6.3.0; Heidelberg Engineering) was used for the SD-OCT assessment. The Spectralis OCT has an acquisition rate of 40,000 A-scans per second. It uses a dual-beam SD-OCT and a confocal scanning laser ophthalmoscope (CSLO) that uses a scanning laser diode with a wavelength of 870 nm and an infrared reference image simultaneously to provide images of ocular structures. The instrument incorporates a real-time eye tracking system that couples CSLO and SD-OCT scanners to adjust for eye motion.

**Figure 1:**
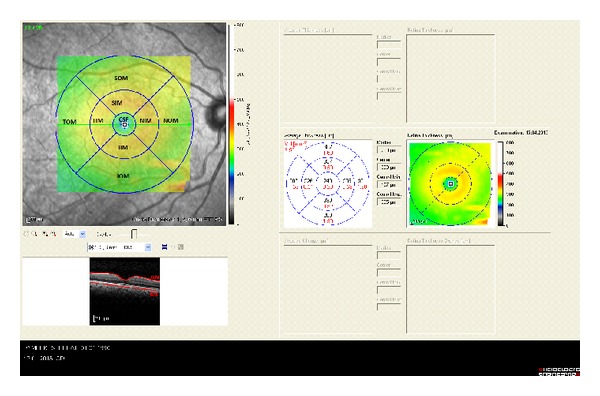
ETDRS template used to derive macular thickness measurements from different regions of a high-density volume scan of the macula. Circles are 1, 3, and 6 mm in diameter. CSF = central subfield; IIM = inferior inner macula; IOM = inferior outer macula; NIM = nasal inner macula; NOM = nasal outer macula; SIM = superior inner macula; SOM = superior outer macula; TIM = temporal inner macula; TOM = temporal outer macula. Control patient.

The macula and peripapillary RNFL examinations were performed using an internal fixator. During the assessments, macular thickness and volume analysis (resolution mode: high speed; scan angle: 30 degrees; size X: 512 pixels (5.8 mm); size Z: 496 pixels (1.9 mm); scaling X: 11.28 m/pixel; scaling Z: 3.87 m/pixel; number of B-scans: 25; pattern size: 20-20 degrees (5.8-5.8 mm), distance between B-scans: 240 m) and peripapillary RNFL thickness analysis (resolution mode: high speed; circle diameter: 3.5 mm; size X: 768 pixels (10.9 mm); size Z: 496 pixels (1.9 mm); scaling X: 14.17 m/pixel; scaling Z: 3.87 m/pixel) were used. The results obtained from the macular scan were classified by region, as shown in Figure 1. We selected the RT map analysis protocol on the Spectralis to display numeric averages of the measurements for each of the 9 subfields, as defined by the Early Treatment Diabetic Retinopathy Study [12].

The average of all points within the inner circle with a 1 mm radius was defined as the central foveal subfield (CSF) thickness. The central point, which is an average of six radial scans at the foveola, was defined as the central point thickness and was recorded for each of the subjects. The following are the abbreviations used: CSF = central subfield; SIM = superior inner macula; TIM = temporal inner macula; IIM = inferior inner macula; NIM = nasal inner macula; SOM = superior outer macula; TOM = temporal outer macula; IOM = inferior outer macula; NOM = nasal outer macula.

**Figure 2:**
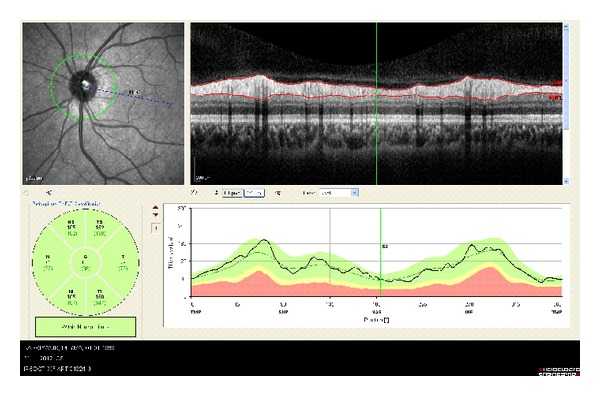
Evaluation of RNFL. Sample retinal nerve fiber layer report provided by the Spectralis device for a retinal nerve fiber layer scan. Numbers directly under each sector name are the individuals mean retinal nerve fiber layer thickness (*μ*m). Control patient.

The peripapillary RNFL thickness parameters that were automatically calculated by the SD-OCT device and divided into regions include temporal quadrant thickness (90 degrees), temporal superior quadrant thickness (45 degrees), nasal superior quadrant thickness (45 degrees), nasal quadrant thickness (90 degrees), nasal inferior quadrant thickness (45 degrees), temporal inferior quadrant thickness (45 degrees), and average thickness (360 degrees) (Figure 2). After the exposures, the noncentered scans and the scans with signal strength <15 dB were excluded from the study.

The method reported previously by Spaide et al. was used to obtain enhanced depth imaging OCT (EDI-OCT) images [13].

The choroid was imaged by positioning a Heidelberg Spectralis (Heidelberg Engineering) close enough to the eye to obtain an inverted image. This image was averaged for 100 scans using the automatic averaging and eye tracking features. Seven sections, each comprised of 100 averaged scans, were obtained in a 5 × 30 degree rectangle encompassing the macula and optic nerve and the horizontal section going directly through the center of the fovea was selected. The resulting images were viewed and measured with the contained Heidelberg Eye Explorer software (version 5.6.3.0; Heidelberg Engineering). The choroid was measured from the outer portion of the hyperreflective line corresponding to the retinal pigment epithelium to the inner surface of the sclera. The measurements of subfoveal choroidal thickness were measured independently by two blinded clinicians (MA and IY), in a masked fashion without knowledge of information of the subjects and the mean values were recorded. Differences in measurement between the interpreters larger than 10% were excluded from the study.

## 3. Statistics

Shapiro-Wilk's test was used and a histogram and* q*-*q* plot were examined to assess the data normality. Levene's test was used to assess the variance homogeneity. A two-sided independent sample* t*-test and one-way analysis of variance was applied to compare the differences between continuous variables. Welch's test was applied when the homogeneity of the variance assumption was violated. Tukey's and Tamhane's* T2* tests were applied for multiple comparisons. Values are expressed as mean ± standard deviation. Analyses were conducted using R 3.0.0 software. Values of *P* < 0.05 were considered to be statistically significant.

## 4. Results

The control group consisted of 40 eyes of 40 volunteers; the individuals were characterized by a mean age of 34.18 ± 10.23 years. Eighty eyes of 80 patients with BD were included in the study. Ocular involvement was present in 40 eyes of 40 patients with BD. The remaining 40 eyes of 40 patients with BD had no ocular involvement. Posterior segment involvement was present in 20 eyes of 20 patients and the remaining had anterior segment involvement. There was no panuveitis in any eyes. The demographical and clinical features of the groups are listed in Table 1.

There was no significant difference between the controls and the groups with and without ocular involvement with respect to age and gender (*P* = 0.75; Table 1).

The mean visual acuity of group 1, BD patients with ocular involvement, was significantly lower than the other two groups (*P* < 0.001).

There was no significant difference between the groups regarding spherical equivalent, axial length, and IOP (*P* = 0.389, 0.083, and 0.296, resp.).

The mean duration of BD, defined as the time elapsed from the diagnosis of BD until the initiation of the present study, was 6.59 ± 3.68 years in group 1 (range: 1–20) and 6.32 ± 4.0 years in group 2 (range: 1–20) (*P* = 0.927).

There was no statistically significant difference in erythrocyte sedimentation rate and C-reactive proteins value between patients in groups 1 and 2 (*P* = 0.51 and 0.36, resp.).

Macular and choroidal thickness analysis, macular volume analysis, and peripapillary RNFL thickness analysis obtained by the SD-OCT are given in Tables 2, 3, and 4, respectively.

Macular thickness in SIM, TIM, IIM, NIM, SOM, IOM, and NOM is significantly thinner in patients with BD than the control group (*P* = 0.05). Furthermore, macular thickness in SIM, TIM, NIM, SOM, IOM, and NOM is significantly thinner in patients with ocular involvement than the controls and patients without ocular involvement.

There was no statistically significant difference in thickness between RNFL analyses of patients with BD and the control subjects. However, BD patients with ocular involvement had statistically significant thinning in NazS, Naz, TempI, and AVE compared with BD patients without ocular involvement (Table 4).

RNF thickness in the nasal part in the subgroup analysis was 77.81 ± 13.92, 73.45 ± 14.85, and 53.75 ± 13.93 in controls, patients without ocular involvement, and patients with posterior segment involvement, respectively (*P* = 0.014).

Although the choroid was measured as being thicker in patients with BD than the control group, it was not statistically significant (*P* = 0.382). In subgroup analysis, even though the choroid was thicker in those with posterior segment involvement than in those without ocular involvement, the difference did not prove to be statistically significant. The thickness of the choroid was 325.41 ± 60.24, 325.13 ± 64.63, and 336.50 ± 10.08 *μ*m in controls, patients without ocular involvement, and patients with posterior segment involvement, respectively (*P* = 0.68).

Because BD patients in our study were in the inactive phase, leakage in most of the patients, according to retinal vascular leakage grading system, was graded 0 or 1. Therefore, we could not find any correlation between FA findings and choroidal, RNFL, and macular thickness.

**Figure 3:**
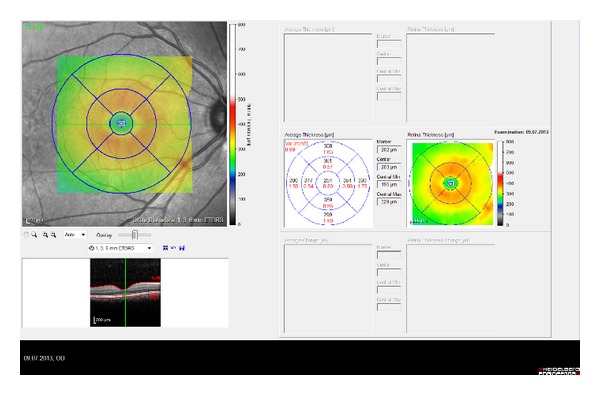
Macular thickness of BD patient with ocular involvement.

**Figure 4:**
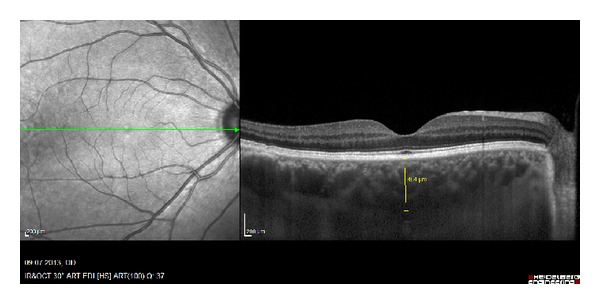
EDI-OCT images of the right eyes of patients having Behçet's disease with ocular involvement. Same patient above.

**Figure 5:**
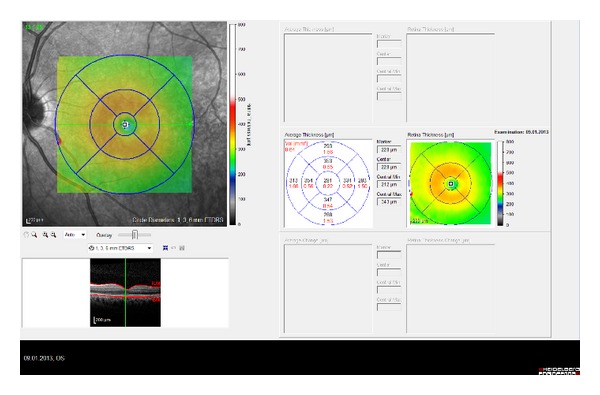
Macular thickness of BD patient without ocular involvement.

**Figure 6:**
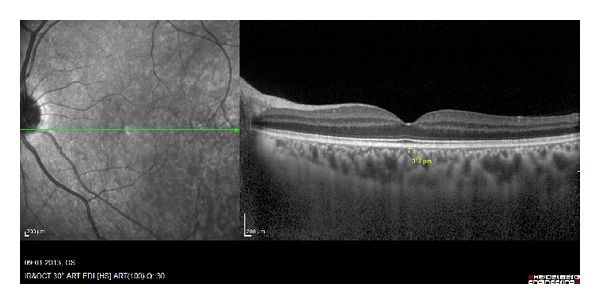
EDI-OCT images of the left eyes of patients having Behçet's disease without ocular involvement. Same patient above.

Macular and choroidal thickness on EDI-OCT images of BD patients with and without ocular involvement is shown in Figures 3, 4, 5, and 6.

## 5. Discussion

Macular thickness analysis with OCT could be useful in diagnosing complications related to posterior uveitis, such as macular edema or macular atrophy. Retinal complications that develop following a uveitis attack may also be assessed via OCT [14].

We found that macular thickness in SIM, TIM, IIM, NIM, SOM, IOM, and NOM was significantly reduced in patients with BD compared with the control group. We did not find any statistically significant difference, except for the thinning in SOM of group 1 and in macular thickness between groups 1 and 2.

Grover and his colleagues [15] found a CSF measurement of 270.2 *μ*m in the normal population. In our study, we found CSF values of 269.31, 266, and 269 *μ*m in the control group, group 1, and group 2, respectively. These results are consistent with the results of study conducted by Grover and his colleagues.

Unoki at al. [16] researched structural changes in the fovea during the remission phase of BD. These authors showed that foveal thickness in the IS/OS (−) eye group was significantly thinner than in the IS/OS (+) eye group. In our study, the group showing ocular involvement also exhibited thinning in some macular areas compared with the group that did not show ocular involvement and the control group (Table 2).

The main pathology in BD is occlusive vasculitis. As a result of recurrent vasculitis, ischemia followed by atrophies occurs in areas fed by the occluded vessels [17]. Also, it has been found that ocular blood flow decreases in BD [18]. Both the inflammation and the decreased blood flow may lead to retinal nerve fiber loss and optic nerve damage. OCT may identify the early stage damage in retinal nerve fiber layers and may provide quantitative measurements of RNFL in different quadrants [19, 20].

Tekeli and Özdemir [17] analyzed disk topography in healthy controls and in BD patients with and without ocular involvement using Heidelberg Retinal Tomography and found that the average disk area, cup area, cup volume, and cup depth in BD patients with and without ocular involvement were significantly smaller than the control group. Furthermore, Şakalar et al. [21] compared normal individuals with BD patients without ocular involvement in terms of their RNFL measurements and found an RNFL thickness of 104.08 ± 8.27 *μ*m on average in the control group and 106.98 ± 9.14 *μ*m in BD patients without ocular involvement. These authors did not find any statistically significant differences in RNFL thicknesses between the groups at all.

In our study, there was no statistically significant difference in thickness between RNFL analysis of patients with BD and the control subjects. However, the BD patients with ocular involvement had statistically significant thinning in NazS, Naz, TempI, and AVE compared with the BD patients without ocular involvement (Table 4). The average RNFL was found to be 100.23 ± 10.01 *μ*m in the control group and 100.74 ± 10.64 *μ*m in the BD patients without eye involvement. Furthermore, in agreement with the study of Şakalar et al., our study did not reveal a statistically significant difference. While the average thickness of the retinal nerve fiber layer was 93.52 in group 1, the average thickness of retinal nerve fiber layer was 100.74 in the group that did not show ocular involvement. The thinning in RNFL in group 1 occurs at approximately the 7% level. We believe that this percentage of thinning may be significant during clinical practices.

Berker and his colleagues [22] found that the mean cup volume, rim volume, cup area, and cup depth were greater in BD patients with mild uveitis compared with BD patients with severe uveitis. The difference in the cup to disk ratio between the two groups was not found to be statistically significant.

In our work, while thinning was observed, in particular in nasal and nasal superior areas of the disk in BD patients, this thinning was more pronounced in BD patients showing ocular involvement. Moreover, statistically significant thinning was found in the average RNFL in ocular BD patients.

The thinning of the nasal disk, which we report, is a novel finding that should be taken into consideration in ischemic and vasoocclusive conditions such as diabetes and BD. Vujosevic et al. [23] reported that choroidal thickness is reduced in diabetic eyes and parallels the appearance and evolution of diabetic retinopathy. The nasal quadrant, in their study, was the most affected area. The nasal quadrant, which the choroid layer supplies blood to, was deteriorated. We believe that this effect may explain why the nasal part of RNFL was significantly reduced in patients with BD. Moreover, Garhofer et al. [24] reported that retinal blood flow was the highest in the temporal inferior quadrant, followed by the temporal superior quadrant, the nasal inferior quadrant, and the nasal superior quadrant, which supports our findings. In order to further clarify this issue, additional studies need to be conducted.

BD is characterized by leukocytoclastic vasculitis that results in the obliteration of the vascular endothelial lumen [3, 4]. Histopathological studies on eyes with BD showed intensive retinal vasculitis and diffuse or focal infiltration of the choroid with inflammatory cells but no choroidal vasculitis [3–5].

The choroid, which constitutes the middle vascular ocular layer between the outermost sclera and the innermost retina, plays an important role in the pathogenesis of many diseases of the posterior segment of the eye. A better understanding of the structure of the choroid is important to furthering understanding of the pathophysiology of many diseases. Adequate visualization of the choroid is still lacking despite advances in imaging technology. Traditional imaging modalities, such as B-scan ultrasonography and ICG, are limited in image resolution and measurement accuracy. EDI-OCT provides many details of the microarchitecture of the posterior choroid and theoretically facilitates an understanding of the choroidal abnormalities underlying various chorioretinal diseases [25–27].

Some studies reported that choroidal thickness decreases with increasing age [28, 29]. Using EDI-OCT, many recent studies have shown that abnormal choroidal thickness may be related to some ocular diseases. A thin choroid has been measured in highly myopic eyes and cases of retinal dystrophy and age-related choroidal atrophy. A thick choroid has been seen in conjunction with central serous chorioretinopathy and Vogt-Koyanagi-Harada disease [26, 29–31].

We measured subfoveal area using EDI-OCT and found that it was 325.41 *μ*m in the control group and 333.61 *μ*m in patients with BD. This difference in choroidal thickness was not found to be statistically significant. Polat et al. [32] measured subfoveal area in healthy individuals and found an average thickness of 287.6 *μ*m. A similar thickness, 265.5 *μ*m, was found by Fujiwara and his colleagues [26] who categorized healthy Japanese individuals into different age groups. The average age in our study was in the range of 34.18–37.92 years and the measurement of this age group was found to be 277.5 *μ*m. In another study, Ikuna and his colleagues [33] focused on the Japanese population and found a choroidal thickness of 354.11 *μ*m.

Atmaca and Sonmez [34] reported hyperfluorescence and/or hypofluorescence, irregular filling of choriocapillaris, choroidal filling defects, and ICG leakage from choroidal vessels on ICGA and suggested that choroidal involvement was present in BD. Iaccarino and his colleagues [7] found an increase in the retinal choroidal thickness in A-scan echographies associated with a normal retinal thickness in OCT in a statistically high percentage of the cases (60%). They suggested that choroidal inflammation was the primary etiological factor during posterior vasculitis in BD.

Kim et al. [8] found an increase in subfoveal choroidal thickness in BD patients with posterior uveitis during the active and inactive phases, which shows a correlation between FA and retinal vascular leakage.

Coskun et al. [9] found a thinning in subfoveal choroid thickness in BD patients with posterior segment involvement. This result suggests that recurrent posterior uveitis may affect choroid circulation and cause atrophy of the choroid.

In our study, although there was an increase in the choroidal thickness of patients with BD compared with controls, the result was not statistically significant (as found by Kim et al.). However, this result might be due to the inactive stage of the disease or the small sample size of our study.

In conclusion, we found thinning in some parts of the macula in BD patients compared with controls, while there was no statistically significant difference in macular thickness between groups either showing or not showing ocular involvement. Although we did not find any statistically significant difference in RNFL between controls and BD patients, we showed a decrease in average RNFL in the group showing ocular involvement compared with the group not showing ocular involvement. An increase in choroidal thickness of BD patients with ocular involvement was assessed but was not statistically significant. BD may be associated with decreased macular thickness measured with spectral-domain OCT.

In the present study, we compared macular, choroid, and peripapillary nerve fiber layer thickness in inactive periods of ocular BD and healthy subjects. Based on the results of our study, macular thickness is thinner in inactive periods of BD. Although we did not find any statistically significant difference in RNFL between controls and BD patients, we showed a decrease in average RNFL in the group showing ocular involvement compared with the group not showing ocular involvement. In conclusion, using OCT during routine follow-ups of BD patients may be beneficial for evaluating ocular involvement.

## Figures and Tables

**Table 1:** Demographic and clinic features of the groups.

Variables	Controls (*n* = 40)	Ocular involvement (group 1 *n* = 40)	Without ocular involvement (group 2 *n* = 40)	*P* value^a^
Age	34.18 ± 10.23	35.95 ± 7.82	37.92 ± 10.48	0.075
Gender (M/F)	20/20	22/18	21/19	0.912
Axial length (mm)	23.46 ± 0.056	23.63 ± 0.51	23.36 ± 0.64	0.083
Spherical equivalent (diopter)	−0.20 ± 0.33	−0.28 ± 0.58	−0.28 ± 0.37	0.389
Visual acuity (decimal)	0.99 ± 0.03	0.83 ± 0.32	0.96 ± 0.08	<0.001
Subfoveal choroid thickness (*μ*m)	325.92 ± 60.03	347.23 ± 68	325.13 ± 64.63	0.146
disease duration (year)	—	6.46 ± 3.58	6.35 ± 3.99	0.927

^a^One-way ANOVA. Values are expressed as mean ± SD. M: male: F: female.

**Table 2:** Thickness of macula in patients with BD and controls.

Variables	Groups			Ocular Involvement		
	Control (*n* = 40)	Behcet (*n* = 80)	*P*	Present (*n* = 40)	Absent (*n* = 40)	*P*
CSF	269.30 ± 20.05	266.76 ± 22.87	0.425	266 ± 23.07	269 ± 20.51	0.606
SIM	348.75 ± 16.48	340.95 ± 19.37	0.004	336.41 ± 18.17	342.05 ± 19.25	0.271
TIM	333.46 ± 14.00	326.41 ± 16.85	0.003	327.73 ± 15.3	326.84 ± 16.26	0.836
IIM	342.57 ± 18.77	336.47 ± 20.80	0.038	336.86 ± 17.96	335.81 ± 26.53	0.870
NIM	348.28 ± 14.12	338.38 ± 22.48	<0.001	338.68 ± 21.68	339.62 ± 21.03	0870
SOM	301.03 ± 15.21	294.23 ± 17.93	0.006	287.95 ± 16.94	297.43 ± 15.31	0.031
TOM	288.47 ± 15.16	283.90 ± 20.09	0.089	279 ± 16.46	286.3 ± 14.42	0.080
IOM	294.44 ± 16.53	287.95 ± 19.26	0.016	285 ± 31.15	289.97 ± 14.83	0.488
NOM	319.07 ± 15.07	310.18 ± 20.10	0.001	305.64 ± 17.84	312.24 ± 21.13	0.224
FOVEA	226.46 ± 24.78	225.59 ± 30.67	0.492	218.18 ± 25.83	230.16 ± 31.2	0.135

Values are expressed as mean ± SD; *n*: number of case; CSF: central subfield; SIM: superior inner macula; TIM: temporal inner macula; IIM: inferior inner macula; NIM: nasal inner macula; SOM: superior outer macula; TOM: temporal outer macula; IOM: inferior outer macula; NOM: nasal outer macula.

**Table 3:** Volumes of macula in patients with BD and control.

Variables	Groups			Ocular Involvement		
	Control (*n* = 40)	Behcet (*n* = 80)	*P*	Present (*n* = 40)	Absent (*n* = 40)	*P*
CSF	0.21 ± 0.02	0.21 ± 0.02	0.376	0.21 ± 0.02	0.21 ± 0.02	0.987
SIM	0.55 ± 0.03	0.54 ± 0.03	0.003	0.53 ± 0.03	0.54 ± 0.03	0.142
TIM	0.54 ± 0.02	0.52 ± 0.03	0.004	0.52 ± 0.02	0.51 ± 0.03	0.746
IIM	0.54 ± 0.03	0.53 ± 0.03	0.013	0.53 ± 0.03	0.53 ± 0.04	0.831
NIM	0.54 ± 0.03	0.52 ± 0.03	0.007	0.53 ± 0.03	0.54 ± 0.03	0.443
SOM	1.6 ± 0.09	1.56 ± 0.09	0.005	1.52 ± 0.1	1.58 ± 0.08	0.018
TOM	1.61 ± 0.11	1.58 ± 0.12	0.071	1.48 ± 0.09	1.52 ± 0.08	0.133
IOM	1.56 ± 0.08	1.53 ± 0.1	0.053	1.54 ± 0.16	1.54 ± 0.08	0.859
NOM	1.61 ± 0.12	1.58 ± 0.12	0.118	1.62 ± 0.1	1.67 ± 0.08	0.019
AVE	0.97 ± 0.04	0.95 ± 0.05	0.004	0.94 ± 0.05	0.96 ± 0.04	0.171

Values are expressed as mean ± SD; *n*: number of case; CSF: central subfield; SIM: superior inner macula; TIM: temporal inner macula; IIM: inferior inner macula; NIM: nasal inner macula; SOM: superior outer macula; TOM: temporal outer macula; IOM: inferior outer macula; NOM: nasal outer macula; AVE: average volume.

**Table 4:** RNFL of patients with BD and controls.

Variables	Groups			Ocular Involvement		
	Control (*n* = 40)	Behcet (*n* = 80)	*P*	Present (*n* = 40)	Absent (*n* = 40)	*P*
T	72.42 ± 14.23	71.21 ± 12.7	0.541	76.86 ± 15.5	72.29 ± 11.22	0.212
Ts	138.65 ± 18.8	133.46 ± 24.18	0.116	132.38 ± 37.45	137.41 ± 19.86	0.518
Ns	112.43 ± 20.87	109.1 ± 20.25	0.274	96.1 ± 14.95	106.53 ± 18.14	0.031
N	73.45 ± 14.85	74.55 ± 13.83	0.603	66 ± 12.77	78.41 ± 13.71	0.002
Ni	114.39 ± 24.06	109.73 ± 25.01	0.202	100.38 ± 27.64	111.91 ± 21.55	0.087
Ti	145.16 ± 23.26	140.92 ± 23.55	0.223	133.05 ± 19.21	147.31 ± 24.83	0.028
G	100.23 ± 10.01	98.13 ± 11.14	0.186	93.52 ± 10.19	100.74 ± 10.64	0.016

Values are expressed as mean ± SD; *n*: number of case; T: Temporal; Ts: Temporal Superior; Ns: Nasal Superior; N: Nasal; Ni: Nasal Inferior; Ti: Temporal inferior; G: average.
